# Missed opportunity for routine vaccination and associated factors among children aged 0–23 months in public health facilities of Jimma Town

**DOI:** 10.1371/journal.pgph.0001819

**Published:** 2023-07-25

**Authors:** Halima Abatemam, Mulumebet Abera Wordofa, Bekelu Teka Worku

**Affiliations:** Department of Population and Family Health, Jimma University, Jimma, Ethiopia; VART Consulting PVT LTD, INDIA

## Abstract

The current recommendation obligates children to receive all vaccines within two years of birth. The Expanded Program on Immunization (EPI) was established in Ethiopia to increase the immunization rate by 10% annually and to reach 100% in 10 years but not been achieved in twenty years. Missed opportunity for vaccination (MOV) is one of the major factors in vaccination coverage. Hence, this study aimed to assess the prevalence of MOV and associated factors in Jimma Town public health facilities. A facility-based cross-sectional study design was employed with a quantitative data collection method. The sample size was calculated using a single population proportion formula. The data were collected through face-to-face interviews, and data extraction methods and analyzed using SPSS version 26. The statistical association was decided at p-value <0.05 with 95% CI, and AOR. A total of 422 children were involved in this study making a 100% response rate. The magnitude of MOV was 39.8% (95%CI: 35–45). Parents/caretakers have not attended formal education (AOR = 4.65, CI:1.64–13.24), residing in rural (AOR = 2.60, CI: 1.35–5.03), poor knowledge about immunization (AOR = 2.61, CI: 1.58–4.30), the child not assessed for vaccination status (AOR = 3.01, CI: 1.65–5.49), and parents/caretakers not seen/heard vaccination message in the last month (AOR = 2.42, CI: 1.40–4.18) were statistically positively associated with the MOV. In conclusion, this study indicated that MOV among the children was high in the study facilities. The researchers recommended stakeholders work on strengthening community awareness creation. Additionally, further study incorporating physician-related factors is also suggested.

## Background

Immunization is a public health important program to reduce the burden of under-five child morbidity and mortality attributed to vaccine-preventable diseases such as measles, diphtheria, influenza, tetanus, pertussis, hepatitis virus, mumps, pneumonia, poliovirus, rotavirus, and others [1, 2]. Immunization saves an estimated 4–5 million under-five child deaths yearly worldwide [3]. From 2000 to 2019, vaccination averted at least 37 million child deaths globally [4].

The World Health Organization (WHO) launched the Expanded Program on Immunization (EPI) in 1974 intending to immunize all children around the world and established a standardized vaccination schedule for six vaccines in 1984: Bacillus Calmette-Guérin (BCG), diphtheria-tetanus-pertussis (DTP), oral polio, and measles [5, 6].

In Ethiopia the immunization program; the EPI was launched in 1980 to reduce mortality and morbidity among children. The EPI was established to increase the immunization rate by 10% annually and to reach 100% in consecutive 10 years [7]. However, this goal was not achieved in 20 years, not only in ten years [8].

Current recommendation obligates, children should receive all vaccines within 2 years of birth: one dose of BCG and oral polio at birth or as soon as possible, three doses of oral polio vaccine (OPV), three doses of Penta-valent, three doses of pneumococcal vaccines, two doses of rotavirus, and Inactivated polio vaccine (IPV) at an interval of 4 weeks from birth, and the first dose of measles at 9-month and the second dose of measles at 15- month old. DTP3 coverage by the age of 12 months is an indicator of immunization program performance [9].

Missed opportunity for routine vaccination (MOV) is when a child who is eligible for vaccination has any contact with health services but does not receive one or more of the vaccine doses on the exit [10]. MOV occurs under two major settings: one is during visits for vaccination and the other is during preventive services such as growth monitoring, nutrition assessments, and visits for curative services [11, 12].

Minimizing MOV reduces disease, death, and disability by increasing immunization coverage and improving the timeliness of vaccination for children [13]. Generally, it helps to improve health service delivery, promotes synergy between treatment services and preventive programs at a health facility level, reduces the number of visits, increases client satisfaction, minimizes costs to the households and health facility, and reduces workload pressures also [10, 13–15].

Despite all its advantages, and the efforts made to increase vaccination coverage, the percentage of children’s full vaccination remains low in Ethiopia [16, 17]. According to the Ethiopian Demographic Health Survey reports only 39.7% and 44% of children were fully vaccinated in 2016 and 2019 respectively [18, 19].

Studies had indicated that MOV was one of the major factors in vaccination coverage in Ethiopia [16, 17]. Hence, assessing the magnitude of MOV and its associated factors could help to produce data that can be used to design appropriate integrated immunization programs and to make an intervention on identified factors in this country. The information generated through this research could also contribute to assisting policymakers to design appropriate programs addressing MOV. Hence, this study aimed to produce updated information on the prevalence of missed opportunities for routine vaccination and associated factors in Jimma Town public health facilities.

## Method

### Study area and period

**Fig 1:**
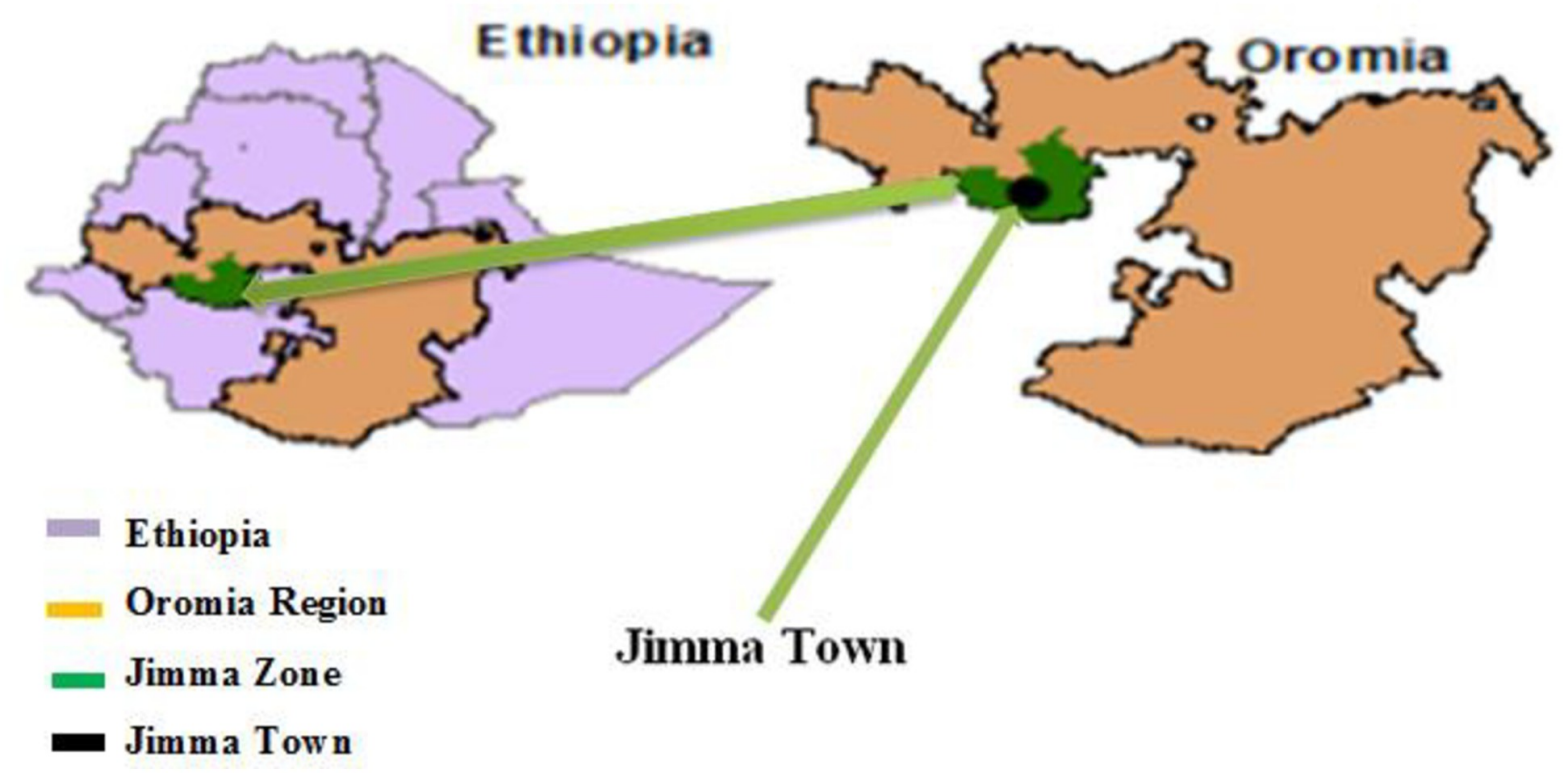
Map of the study area adapted from relevant literature (http://www.planiglobe.com).

The study was conducted in two public hospitals and two public health centers in Jimma Town: Jimma University Medical Center, Shanan Gibe General Hospital, Jimma Health Center, and Higher 2 Health Center. Jimma town is located 353 km from Addis Ababa, the capital city of Ethiopia to the Southwest (Fig 1) [20]. The study was conducted from September 15 to October 16, 2022 All the participant recruitment, interviews, and medical record card reviews were done within these specific days.

#### Study design

An institution-based cross-sectional study design was employed with a quantitative data collection method.

### Population for the study

All eligible children aged between 0–23 months and attending immunization clinic, pediatrics clinic, and first postnatal care unit during the study period at the selected facilities were considered for the study. However, children who were severely ill and who started vaccination without documented vaccination history (either from a vaccination card or health facility card) were excluded during the data collection.

The sample size was determined using the single population proportion formula [21], considering the 49.1% prevalence of MOV which was taken from a study done in Wolikte health center, Ethiopia [16], with a 0.05 margin of error, and 95% confidence level at Zα/2 = 1.96. Accordingly, $\text{n}=\frac{{\left(Z_{\alpha /2}\right)}^{2}p\left(1-p\right)}{d^{2}}=\frac{{\left(1.96\right)}^{2}\;\times 0.491\times 0.509}{{\left(0.05\right)}^{2}}=384$, where n = the minimum required sample size for this study. Adding 10% for the expected non-response rate, the final sample size becomes 422. The calculated sample was distributed by proportional allocation to the size to all the selected public health facilities. Finally, the study participants were included by consecutive sampling technique once screened for the inclusion criteria.

### Ethics statement

This study was approved by the institutional review board of Jimma University and Ethical clearance was obtained with JUIH/IRB 124/22 reference. All methods were carried out per relevant guidelines and regulations of the university’s Institutional Review Board. Study participants were provided comprehensive information on the nature, objectives, benefits, and their right to refuse and assured confidentiality. Oral informed consent was obtained from study participants. The authors had no access to information that could identify individual participants during or after data collection. During the data collection, all data collectors and the study participants adhered to the COVID-19 precautionary methods.

### Data collection tools, personnel, and procedures

A semi-structured questionnaire for face-to-face interview-administered data collection and a checklist for data extraction through document review were used. The tools were adapted from the revised WHO missed opportunity assessment tools and other related literature [12, 22, 23]. The questionnaires for a face-to-face interview were prepared in English, and translated into the local languages (Afaan Oromo and Amharic). Before the actual data collection, a pretest was conducted at Seka Hospital.

Two supervisors and six data collectors were involved in data collection taking two days of training on the content of the questionnaire, issues of confidentiality, ethical conduct of research, and data-collection procedure/techniques.

### Measurements

#### Missed opportunity for vaccination

Children aged between 0–23 months who come to the health facility to get health services with a recommended time range of immunization and who do not get any of the vaccines (one or more) without contraindication for that particular vaccine on the day of the assessment is considered as a MOV. The MOV was measured based on the child’s date of birth, interview date and according to the Ethiopian national immunization schedule which has a total of 6 contact times: at birth, 6^th^ weeks, 10^th^ weeks, 14^th^ weeks, 9 months, and 18 months up to 23 months of life [24].

#### Knowledge of immunization

Participants were asked eight knowledge-related questions, with an expected minimum score of zero and a maximum of twenty one. A score of 1 was given if the parents/caretakers answered the given question correctly and 0 if not. Those who scored the mean and above were categorized as having good knowledge and those who scored below the mean were considered as having poor knowledge [25, 26].

#### Attitude towards immunization

Measured by the attitude assessing tool of eight Likert-scaled question items. Each item of the question has 5 points ranging from 1 (Strongly disagree) to 5 (Strongly agree). Based on a five-point Likert scale for each statement, participants could choose between five possible response categories: “Strongly agree”, “agree”, “neutral”, “disagree” and“Strongly disagree”. Participants who scored mean and above were considered to have a favorable attitude and those who scored below the mean to have an unfavorable attitude [25, 26].

#### Contraindication for vaccination

The Previous reaction to a particular vaccine such as convulsion, anaphylaxis, shock, and encephalopathy soon after or within three days of DPT vaccine injection or children who developed anaphylaxis following measles immunization or convulsion following hepatitis B immunization or if the child is symptomatic HIV/AIDS infected.

#### Caregiver

Any person who adopted the child, or is otherwise taking care of the child, such as an aunt, or grandmother.

### Data analysis

Filled questionnaires were checked for completeness, cleaned, coded, and entered into EPI data statistical software version 4.6.1 and exported to SPSS version 26 for analysis. Frequencies, proportions, and summary statistics were used to describe the study population with relevant variables. Bivariate analysis was employed to identify candidate variables for multivariable analysis. Variables found to have p-values < 0.25 in bivariate analysis were entered into the multivariable logistic regression. Finally, a significant association was determined at p-value <0.05 considering the Adjusted Odds Ratio with 95% CI for the strength of the associations. Hosmer and Lemeshow test was used to check the fitness of the model and it was 0.802. Multicollonirety was checked and all variables have VIF < 2.

## Result

### Socio-demographic characteristics of the children and the parents/caregivers

A total of 422 parents/caregivers who visited the health facility with their children responded to the questions making a 100% response rate. More than half of the 220(52.1%) children were males. The mean age of the children was 6.48 (SD = ±5.182) months (Table 1).

**Table 1:** Socio-demographic characteristics of the study children and parents/caregivers.

Variables	Category	Frequency(%)
Age of the child	0–11	324(76.8)
	12–23	98(23.2)
Relationship of a person who brings the children	Mother	412(97.6)
	Father	8(1.9)
	Caregiver	2(0.5)
Place of the child’s birth	Home	29(6.9)
	Health facility	393(93.1)
Children whose mothers attended at least one ANC	Yes	409(96.9)
	No	13(3.1)
Educational status of the parents/caregivers	Not attended formal education	122(28.9)
	Primary (1–8) school	159(37.7)
	Secondary (9–12) school	100(23.7)
	Collage and above	41(9.7)
Occupational status of the parents/caregivers	House-wife/farmer	260(61.6)
	Merchant	77(18.3)
	Daily laborer	25(5.9)
	Employee (Government/private)	35(8.3)
	Student	25(5.9)
Marital status of the parents/caregivers	Single	4(0.9)
	Married	389(92.2)
	Divorced/separated	24(5.7)
	Widowed	5(1.2)
The parents’/caregivers’ residence	Urban	346(82.2)
	Rural	76(17.8)

### Parents/caregivers related factors

The mean score of parents’/caregivers’ knowledge of child immunization in this study was 9.44. The finding shows that more than half 224 (53.1%) of the participants had poor knowledge having less than the mean score. Regarding the attitude toward immunization, more than half 261(61.8) of the participants had a favorable attitude (Table 2).

**Table 2:** Parents /caregivers related factors of missed opportunity for routine vaccination.

Variables	Category	Frequency	Percent
Knowledge about immunization	Good	198	46.9
	Poor	224	53.1
Attitude towards immunization	Favorable attitude	261	61.8
	Unfavorable attitude	161	38.2
Having the decision-making power to vaccinate the child	Father	33	7.8
	Mother	46	10.9
	Another relative	2	0.5
	The joint decision of the father and mother	341	80.8

### Missed opportunity for routine vaccination

**Fig 2:**
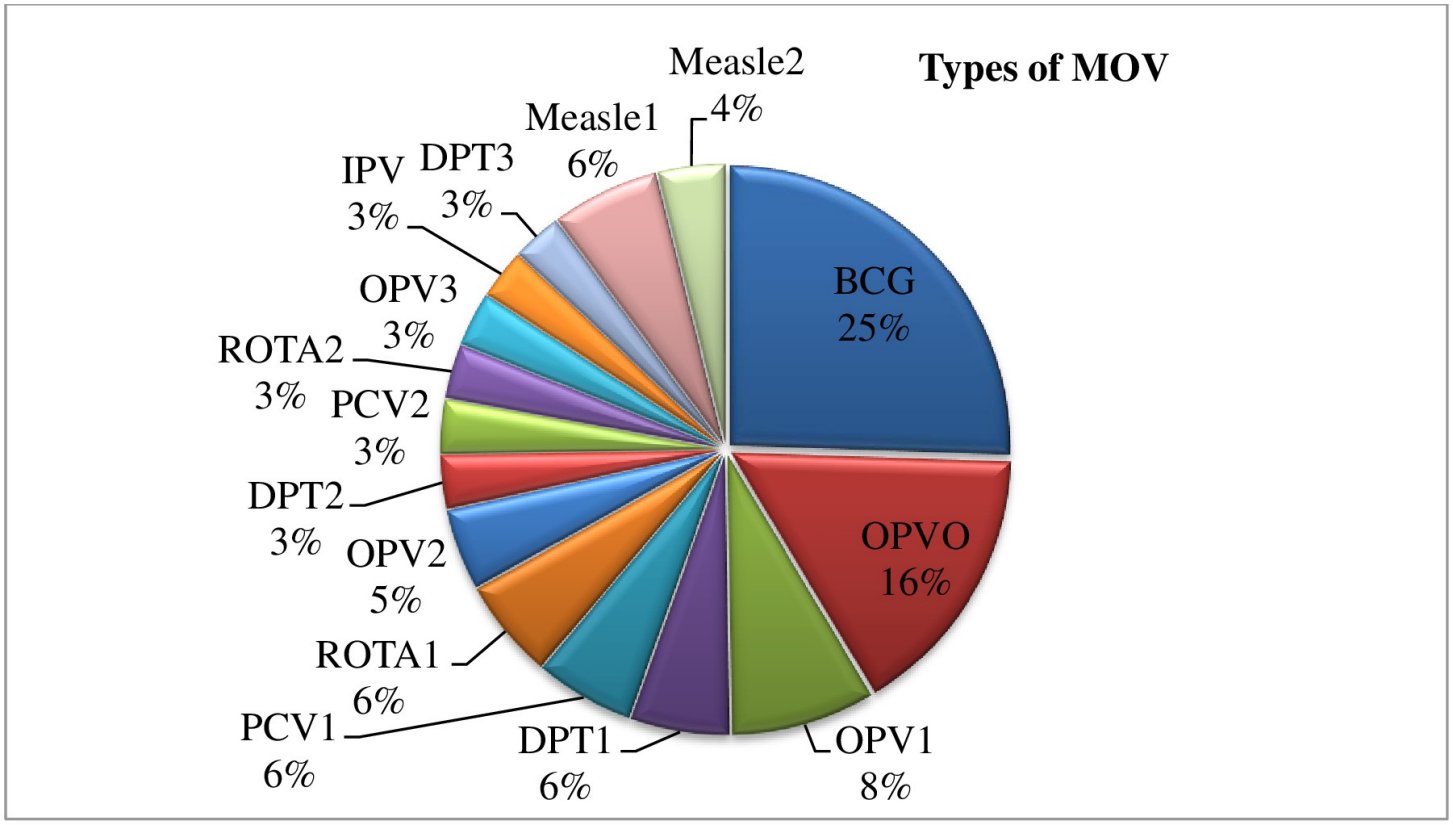
Missed opportunity per vaccine for routine vaccination among study participants in the study area.

The total of missed opportunities for routine vaccination among the study children was 168 (39.8%) [95% CI: 35%-45%)]. The mostly missed vaccines were: 98 (23.2%) BCG, 61 (14.5%) OPV 0, 32 (7.6%) OPV1, and 24 (5.4%) Measle1 (Fig 2).

### Reported reasons for the missed opportunity for routine vaccination

**Fig 3:**
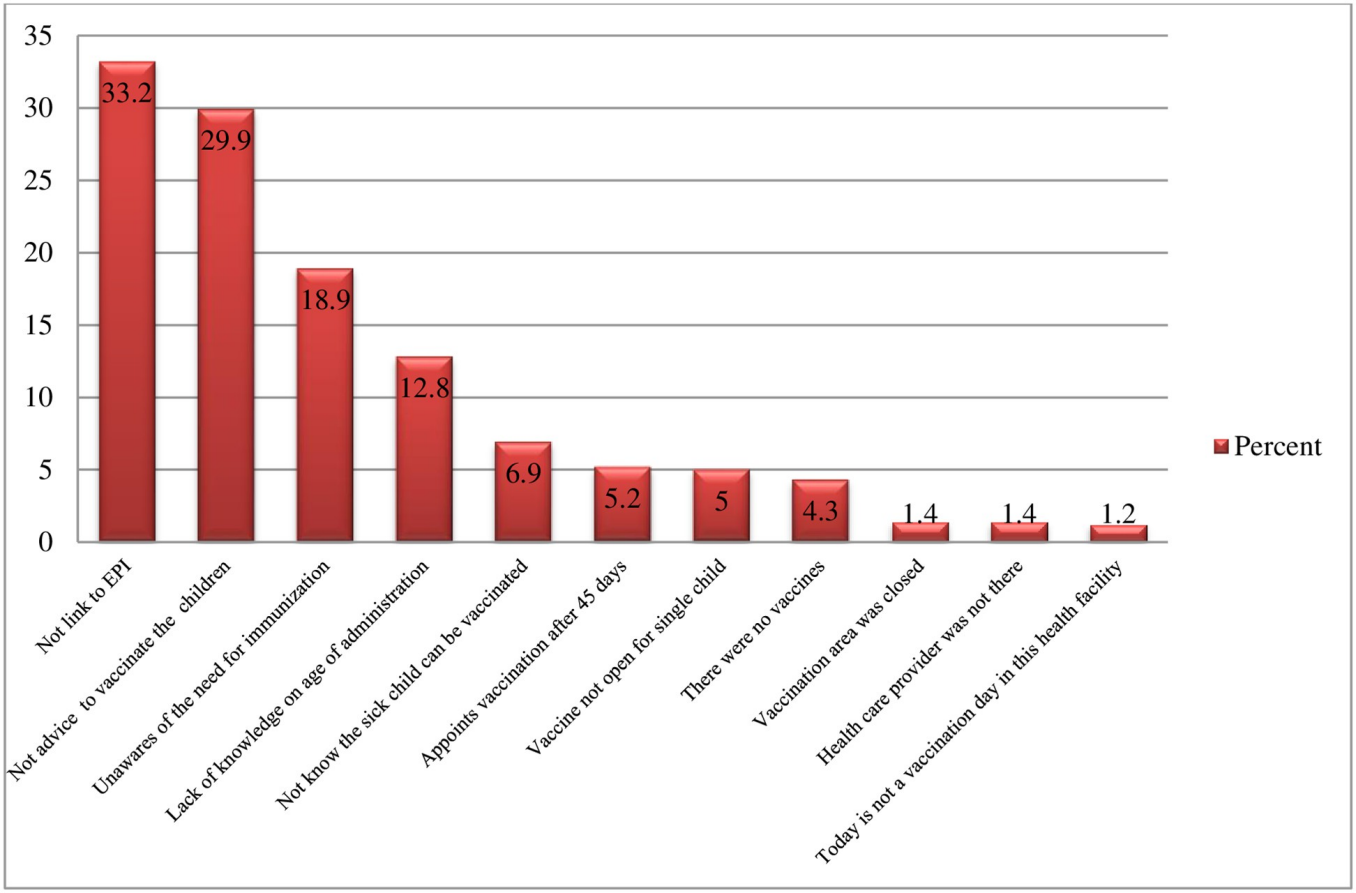
The reported reason for the MOV among the children aged 0–23 months in the selected public health facilities in Jimma Town.

The main reported reason by parents/caregivers for not vaccinating the children on the day of the assessment was not linked to the EPI unite 140 (33.2%) followed by not being advised to vaccinate the children 126 (29.9%) (Fig 3).

### Factors associated with a missed opportunity for routine vaccination

In this study, the odds of MOV among the children whose parents/caregivers did not attend a formal education was 4.65 times (AOR =, 4.65 95%CI: 1.64–13.24) more likely higher compared to those who attended a formal education. The odds of MOV among the rural resident children was 2.60 times (AOR = 2.60, 95%CI: 1.35–5.03) more likely higher than the children living in the urban. Furthermore, the odds of MOV among children whose parents/caregivers had poor knowledge of immunization was 2.61 times (AOR = 2.61, 95%CI: 1.58–4.30) more likely higher than the children whose parents/caregivers had good knowledge. The odds of MOV among the children not screened for vaccination status was 3.01 times (AOR = 3.01, 95%CI: 1.652–5.490) more likely higher than the children screened for their vaccination status. Moreover, the odds of MOV among the children whose parents/caregivers had not seen or heard vaccination messages from different sources of information in the last month was 2.42 times (AOR = 2.42, 95%CI: 1.401–4.175) more likely higher than those whose parents/caregivers had seen or heard vaccination message in the last one month (Table 3).

**Table 3:** Bivariate and multivariable logistic regression output for the factors associated with MOV among children 0–23 months at public health facilities in Jimma Town.

Variables	Categories	Missed opportunity		COR (95%CI)	AOR (95%CI)
		Yes (%)	No (%)		
Birth order	1	51(37.00)	87(63.00)	1	1
	2–3	69(36.50)	120(63.50)	0.98(0.62–1.55)	1.04(0.59–1.84)
	4+	48(50.50)	47(49.50)	1.74(1.03–2.96)	0.69(0.33–1.43)
Educational status	No formal education	83(68.00)	39(32.00)	6.59(2.94–14.80)	4.65(1.64–13.24)*
	Primary	48(30.20)	111(69.80)	1.34(0.61–2.95)	0.82(0.31–2.13)
	Secondary	27(27.00)	73(73.00)	1.15(0.00–2.65)	0.91(0.34–2.47)
	Collage and above	10(24.40)	31(75.60)	1	1
Marital status	Married	147(37.80)	242(62.20)	1	1
	Single/divorced/Separated/widowed	21(63.60)	12(36.40)	2.88(1.38–6.03)	3.81(1.51–9.64)
Place of residence	Urban	114(32.90)	232(67.10)	1	1
	Rural	54(71.10)	22(28.90)	4.99(2.90–8.61)	2.60(1.35–5.03) *
Knowledge	Good knowledge	51(25.80)	147(74.20)	1	1
	Poor knowledge	117(52.20)	107(47.80)	3.15(2.09–4.76)	2.61(1.58–4.30) **
Attitude	Favorable attitude	92(35.20)	169(64.80)	1	1
	Unfavorable attitude	76(47.20)	85(52.80)	1.64(1.10–2.45)	1.43(0.87–2.36)
vaccination status not Screened	Yes	33(18.90)	142(81.10)	5.19(3.29–8.17)	3.01(1.65–5.49)**
	No	135(54.70)	112(45.30)	1	1
Information seen or heard in the last months	Yes	49(23.40)	160(76.60)	1	1
	No	119(55.90)	94(44.10)	4.13(2.72–6.29)	2.42(1.40–4.18) *

*statistically significant association at p-value <0.05,

**statistically significant association at p<0.001,

COR = Crude odds ratio, AOR = Adjusted odds ratio.

## Discussion

Neonatal and child health is a crucial public health importance in the world. However, the quality of neonate and child health care is poor in low and middle-income countries and persisted impacting global neonates’ and children’s health [27, 28]. There was an increasing trend of under-five child mortality in the aftermath of the Millennium Development Goal (MDG) in Ethiopia, even though the child health-related goal was achieved ahead of the due date [29]. Both community-based and facility-based child health care use is still not satisfactory and it needs more attention and activities in this country [30, 31]. This study identified the prevalence of facility-based MOV among children aged 0–23 months, reported possible reasons for the MOV, and revealed significantly associated factors with the MOV in Jimma Town public health facilities.

The prevalence of MOV among the children who visited public facilities was 39.80% (95% CI: 35%-45%) in this study. This result is consistent with the result from a study conducted in Sudan 35% [32] and the Dominican Republic 42.8% [33]. According to these findings, the prevalence of MOV was high among the children which in turn indicates the low vaccination percentage in all the study facilities. This could be because health service utilization and vaccination are basically low in less developed countries and it is aggravated since the emergency of COVID-19 [34–36].

Conversely, the prevalence of MOV in this study was low compared to the prevalence (74.9%) from a study conducted in the Gozamen district, Ethiopia [17] and Kenya (75%) [37]. The reason for this variation might be due to the difference in study time as the missing vaccination can be reduced over time due to different interventions. In line with this, systematic review and meta-analysis concluded that integration of immunization with other services improved childhood immunization coverage in low and middle-income countries (LMIC) [38].

On the other hand, the MOV finding in this study was higher than the findings of the study conducted in Cape Town 14.1% [39]. The difference might be due to the poor screening (58.53%) and linkage practice of the physicians in the current study facilities. Moreover, the variation might be due to the difference in the status of the study settings where half of the study facilities in this study are referral facilities having high client flows and creating a high workload which may reduce child screening for vaccination status. Evidence had confirmed that screening children for vaccination during any contact with health services improves immunization coverage by reducing missed opportunities for vaccinations [40]. However, the screening practice was low (58.53%) in this study. Furthermore, research indicated that a high volume of patients and poor quality of health care are common in Ethiopian public health facilities [41].

In this study, the children from non-educated families were more at risk for MOV. The result identified the odds of MOV among the children whose parents/caregivers did not attend formal education was about five times more likely to be higher than those whose parents/caregivers attended formal education. This finding is consistent with a study done in Gozamen [17], cape town south Africa [39], and Zhejiang province, east China [42]. This might be because educated mothers could have better knowledge, understanding, and decision-making practice on different issues of the child’s health including asking for a missed vaccine for their children. In support of this, study finding from Ethiopia and Pakistan showed that maternal education and empowerment had a significant positive effect on child polio vaccination uptake [43, 44].

The study children who were residing in rural were less likely to get vaccinated than urban residents. This result is in line with the finding of the study from India [45]. The lesser chance of MOV among urban children might be for the reason of urban residents are more access to information and education which may determine their ability to do efforts to ask for the service for their children during a facility visit. Further analysis of Ethiopian demographic and health survey showed women who were residing in urban, and who attended primary, or secondary school had significantly associated with full immunization of their children [43].

The odds of MOV among the children whose parents/caregivers had a poor knowledge of immunization was nearly three times more likely compared to those whose parents/caregivers had a good knowledge of immunization. This result indicated parents’/caregivers’ knowledge of vaccination had contributed to reducing MOV for children. This finding is consistent with the findings of research conducted in the Welkite health facilities [16], India [45], China [42], and Juba Teaching Hospital [46]. This association could be justified as, having poor knowledge of immunization like contact time or how many schedules remain for the child to be fully immunized, recommended timeline or immunization schedule for vaccination, and vaccine-preventable disease could increase the risk of MOV. A systematic review in Africa established that childhood immunization uptake can be decreased by maternal knowledge, maternal attitude, and self-efficacy [47].

Similarly, the chance of MOV among the children who did not screen status for their vaccination was more than those who were screened. This result is in line with other study results in India [46], Dominican Republic [33], and Nigeria [48]. This might be because the children cannot be vaccinated if not screened for their vaccination status. The Polio vaccine screening checklist and guideline recommends child screening for vaccination status and contradiction by asking parents/guardians to deliver the service [49].

Additionally, getting a vaccination message was a useful tool among the study participants to reduce the likelihood of MOV among the children. The study indicated that the odds of MOV among the children whose parents/caregivers had not seen or heard vaccination messages from a different source in the last month was around two times more likely than the children whose parents/caregivers had seen or heard vaccination message in the last month. This finding is consistent with the findings of studies conducted in Welkite [16], Juba [46], and Cape Town, South Africa [39]. The significant association could be due to the importance of the vaccination information to use the service at any time. Literature indicated the high risk of MOV among children if parents/caregivers do not have its information [46].

## Conclusion and recommendation

This study indicated that MOV among children aged 0–23 months was high in the study facilities. BCG, OPV 0, and measles1 were the most missed vaccines while the major reasons for missing these vaccines were not linked to the EPI unit followed by not being advised. On the other hand, parents’/caregivers’ education, poor knowledge of immunization, screening the child’s vaccination status, and hearing/seeing vaccination messages were factors that had significant statistical associations with the MOV.

The researchers recommended all stakeholders work on reducing vaccine stockout and strengthening community awareness creation. Further research shall be conducted to identify the physician and facility-related obstacles to child vaccination once the children arrived at a health facility.

### Strength of the study

This study focused on child vaccination which is always a critical service to children and it is a part of key activities to achieve the sustainable development goal and the global agenda. However, this study is not without limitations as it was implemented at the facility level and the result cannot account for the children within communities.
